# In Vitro Toxicity Assessment of *Cortinarius sanguineus* Anthraquinone Aglycone Extract

**DOI:** 10.3390/jof10060369

**Published:** 2024-05-21

**Authors:** Johanna Yli-Öyrä, Mikko Herrala, Harri Kovakoski, Eevi Huuskonen, Peppi Toukola, Riikka Räisänen, Jaana Rysä

**Affiliations:** 1 School of Pharmacy, University of Eastern Finland, 70210 Kuopio, Finland; johanna.yli-oyra@uef.fi (J.Y.-Ö.); mikko.herrala@uef.fi (M.H.); harri.kovakoski@gmail.com (H.K.); eevi.huuskonen@gmail.com (E.H.); 2 Craft Studies, University of Helsinki, 00014 Helsinki, Finland; peppi.toukola@helsinki.fi (P.T.); riikka.raisanen@helsinki.fi (R.R.)

**Keywords:** allergy, anthraquinone, *Cortinarius sanguineus*, cytotoxicity, emodin, genotoxicity, in vitro, natural dye, oxidative stress

## Abstract

Biocolourants could be a sustainable option for dyes that require fossil-based chemicals in their synthesis. We studied the in vitro toxicity of anthraquinone aglycone extract obtained from *Cortinarius sanguineus* fungus and compared it to the toxicity of its two main components, emodin and previously studied dermocybin. Cell viability, cytotoxicity, and oxidative stress responses in HepG2 liver and THP-1 immune cell lines were studied along with skin sensitisation. In addition, genotoxicity was studied with comet assay in HepG2 cells. Cellular viability was determined by MTT, propidium iodide, and lactate dehydrogenase assays, which showed that the highest doses of both the aglycone extract and emodin affected the viability. However, the effect did not occur in all of the used assays. Notably, after both exposures, a dose-dependent increase in oxidative stress factors was observed in both cell lines as measured by MitoSOX and dihydroethidium assays. *C. sanguineus* extract was not genotoxic in the comet assay. Importantly, both emodin and the extract activated the skin sensitisation pathway in the KeratinoSens assay, suggesting that they can induce allergy in humans. As emodin has shown cytotoxic and skin-sensitising effects, it is possible that the adverse effects caused by the extract are also mediated by it since it is the main component present in the fungus.

## 1. Introduction

Anthraquinones are the second-largest group of synthetic dyes after azo dyes [1]. In history, red-producing anthraquinone dyes were obtained from natural sources, such as madder (*Rubia* spp.) and cochineal (*Dactylopius* spp.), but currently, they are produced mainly synthetically. However, they are derived from fossil sources from which the world is trying to shift away as a part of the green transition [2]. Therefore, more sustainable sources and production methods for dyes are needed. On an industrial scale, natural dyes are used to a minor extent.

*Cortinarius sanguineus* (blood-red webcap) is a fungus able to produce at least fifteen different anthraquinones [3]. In the fungi collected from the Savo region in Finland, the dye yield was 6% of dry weight (dw), and the most abundant anthraquinones were emodin (63% dw), dermocybin (31% dw), and dermorubin (4% dw), which produce yellow-to-red shades [4,5]. The minor part (2%) of the fungal anthraquinones consisted of physcion, endocrocin, dermolutein, 5-chloro-dermorubin, as well as 7- and 5,7-chloro-emodin derivatives. The identification of these minor compounds has been described earlier [4,5]. Dermocybin, dermorubin, and emodin have proven to be suitable colourants for several materials, and they also work in the waterless dyeing of textiles [6,7,8,9,10]. However, the production of dyes obtained from natural sources is complicated by several factors, such as seasonal variability, which leads to unreliable supply. To tackle this challenge, gene technology methods for producing natural dyes are being developed. For example, *Saccharomyces cerevisiae* and *Escherichia coli* could be used as hosts for dye-producing genes [11,12,13,14].

Currently, however, fungal dyes are most often used as mixtures, especially by crafters and small-scale businesses, as this is how they exist in nature. It is also possible, even though difficult, to separate the compounds to be used as single dyes [15]. Crafters do not have access to the separation methods, and they are costly, which makes the process less attractive. Furthermore, natural origin does not guarantee the safety of a substance. Dyes are often used in textiles and clothes, posing a risk for contact dermatitis, allergy, or other harmful effects. Various toxicological properties have been observed in natural anthraquinones, including genotoxicity, such as mutagenicity and chromosomal aberrations, as well as kidney toxicity [16,17,18]. However, the information is scarce, and more safety studies on natural dyes are needed. The small-scale dyers and workers at businesses will be exposed to natural anthraquinones and their mixtures, but as stated, information about their safety is still limited.

In our previous study, we determined the cellular toxicity, oxidative stress, mutagenicity, and skin sensitisation potential of *C. sanguineus* dyes dermocybin and dermorubin as pure compounds [7]. Both were non-mutagenic and did not induce skin sensitisation in a gene activation pathway studied with KeratinoSens assay [19]. Also, their cytotoxicity was non-existent or low, even though they increased oxidative stress in the two studied human cell lines. Instead, emodin, the most abundant anthraquinone in *C. sanguineus*, is known to be mutagenic after metabolic activation in the Ames test [20,21] and suspected to be a reproductive toxicant [22], among other effects [18]. In a micronucleus assay conducted with a sea crustacean, *Parhyale hawaiensis*, emodin, along with its chemically modified derivatives, increased the formation of micronuclei, which is a sign of genotoxicity [21].

In the present study, we set out to investigate the in vitro toxicological effects of an anthraquinone extract—namely the aglycone fraction of *C. sanguineus* extract—on cellular toxicity, genotoxicity, and skin sensitisation potential in human cell lines in order to compare the toxicity of an anthraquinone mixture to the toxicity of single dyes. Because the two main components of the fraction are emodin and dermocybin, we also studied the toxicological properties of emodin in the same experimental setting previously used for dermocybin [7]. Finally, we compared the toxicity data of these two major anthraquinones with the results of the extract.

## 2. Materials and Methods

### 2.1. Fungal Anthraquinones

Anthraquinones were isolated from the *C. sanguineus* fungal bodies, and emodin was separated from the extract using multiple liquid–liquid partition as described before [4,5]. Its purity (98.3%) and chemical structure were confirmed by HPLC-DAD-MS and NMR (^13^C and ^1^H) spectroscopy in our previous study [7].

The fungal bodies of *C. sanguineus* were collected from a spruce forest near Kuopio (62°53033″ N, 027°40042″ E), Finland. The mushrooms were used fresh, and the extraction was carried out as described by Hynninen et al. [4]. Fraction 1, which contains anthraquinone aglycones, was used in this study (referred to as aglycone extract hereafter).

For chemical analysis, HPLC-grade methanol (MeOH) and acetonitrile (ACN) were obtained from Honeywell Riedel-de Haën (Charlotte, NC, USA), and formic acid (≥99%) (HCOOH) from VWR International (Radnor, PA, USA). Type 1 water (18.2 MΩ·cm) was used.

The *C. sanguineus* HPLC sample was prepared by dissolving 10 mg of the aglycone extract in 10 mL of MeOH and filtering through 0.22 µm PTFE syringe filters prior to analysis. The HPLC-DAD-ESI-MS/MS analysis was performed with HP Agilent 1100 HPLC system (Agilent Technologies, Santa Clara, CA, USA) equipped with a degasser, a binary pump, an autosampler, and a DAD detector and connected to a Bruker Esquire 3000 plus (Bruker, Billerica, MA, USA) quadrupole ion trap mass spectrometer (MS) equipped with an electrospray ion source (ESI).

Separations were carried out with Gemini C18 column (15 mm × 4.6 mm, 3 μm) (Phenomenex, Torrance, CA, USA) fitted with SecurityGuard C18 guard column using (A) ACN with 0.1% HCOOH (*v*/*v*) and (B) water with 0.1% HCOOH (*v*/*v*) as eluents. Gradient elution was performed at 0–4 min, 10% A; 10 min, 40% A; 55 min, 60% A; and 60 min, 90% A. Flow rate was 0.5 mL/min, and column temperature was 30 °C.

Wavelengths of 254, 285, and 430 nm were monitored with the DAD detector. Positive ionisation-mode MS and negative ionisation-mode MS/MS analysis were employed with the ESI-MS detector. The MS conditions included ionisation capillary voltage at 4.2 kV, drying gas (N_2_) temperature at 300 °C, and nebuliser gas (N_2_) pressure at 30 psi. Full scan mass spectra were measured in a range of *m/z* 50–750.

### 2.2. Cell Culture and Exposures

Human HepG2 liver and human acute monocytic leukaemia THP-1 cells were used to determine the cytotoxicity, genotoxicity, and reactive oxygen species (ROS) production of emodin and the aglycone extract. The dyes were also tested for skin sensitisation potential using KeratinoSens™ assay, which utilises assay-ready keratinocytes. All the cells were maintained in 75 cm^2^ cell culture flasks (VWR, Radnor, PA, USA) in a humidified incubator (MCO-170M-PE, PHC Corporation, PHCbi, Tokyo, Japan), with 5% CO_2_ and a temperature of 37 °C.

HepG2 cells were cultured in Dulbecco’s modified Eagle medium containing 1.0 g/L glucose (DMEM, Gibco, Paisley, UK), supplemented with 10% foetal bovine serum (FBS, Merck, Darmstadt, Germany), 1% L-glutamine (Gibco), 100 U/mL penicillin, and 100 µg/mL streptomycin (Lonza Group Ltd., Basel, Switzerland). The cells were detached after trypsinisation (0.05% Trypsin-EDTA (Gibco) in Dulbecco’s phosphate-buffered saline (DPBS)), and 100,000 cells were seeded in 48-well plates (Thermo Fischer Scientific, Waltham, MA, USA) in 500 µL DMEM per well for toxicity assays, and 150,000 cells were seeded per well in 1 mL of medium in 12-well plates for the comet assay 24 h prior to exposure.

THP-1 cells were cultured in RPMI-1640 medium (HEPES, no glutamine, Gibco), supplemented with 10% FBS performance plus (Gibco), 1% L-glutamine (Gibco), 100 U/mL penicillin, and 100 μg/mL streptomycin (Lonza Group Ltd.). In the experiments, cells were seeded onto 48-well plates, with 175,000 cells in 500 µL medium per well. The cells were differentiated into adherent macrophages by adding 50 nM phorbol 12-myristate 13-acetate (PMA, Sigma-Aldrich, Darmstadt, Germany) into the growth medium. PMA medium was changed on the following day. After 48 h preincubation, exposures were initiated.

In all experiments, dyes were dissolved into dimethyl sulfoxide (DMSO, Sigma-Aldrich). Exposure concentrations were prepared by diluting the dyes with cell culture medium, to 0.03–100 µg/mL of aglycone extract and 0.01–30 µg/mL of emodin. The highest exposure concentration was chosen so that the highest DMSO concentration would be 0.1% or 1% for aglycone extract, depending on the assay, and 1% for emodin. Accordingly, 0.1 or 1% DMSO was used as a vehicle control. The cells were exposed to the colourants for 24 h, except in the skin sensitisation assay, where the exposure time was 48 h. Each test was conducted 3–4 times, and there were 3–4 parallel samples per concentration. Positive controls were as follows: 0.1% Triton X-100 (Sigma-Aldrich) was used in the cell viability and cytotoxicity assays, 200 µM of menadione (Sigma-Aldrich) was utilised in the oxidative stress studies, 15.625–250 M of ethylene glycol dimethacrylate (EGDMA, acCELLerate GmbH, Hamburg, Germany) was used in the skin sensitisation assay, and in the comet assay, 40 µg/mL of methyl methane sulfonate (MMS, Sigma-Aldrich) was used.

### 2.3. Cell Viability and Cytotoxicity

After 24 h exposure, cell viability was assessed with MTT (3-(4,5-dimethylthiazol-2-yl)-2,5-diphenyltetrazolium bromide) assay as described earlier [7]. In MTT test, living cells transform MTT into purple formazan crystals, which are dissolved, and absorbance is measured to assess the viability of the cells. Briefly, the exposure medium was removed, replaced with MTT-containing (0.5 mg/mL) medium and incubated for 2 h at 37 °C. Then, the MTT medium was replaced with sodium dodecyl sulphate—dimethylformamide buffer (SDS-DMF, pH 4.7) to dissolve the crystals, and the well plate was placed on an orbital shaker at room temperature for 35 min. Absorbance was measured with a microplate reader at 570 nm (Hidex Sense 425–301 microplate reader, Hidex, Turku, Finland). The results were then expressed in relation to the control to indicate the level of viable cells.

Further, apoptotic and necrotic cytotoxicity was measured using the lactate dehydrogenase (LDH) and propidium iodide assays following the test outline of our previous research article [7]. Damaged cells release LDH, and it can be measured from exposure medium collected after 24 h of dye exposure. The LDH assay mixture (Sigma-Aldrich) was prepared and added to the 96-well plate, which was incubated for 20 min in the dark at room temperature before measuring the absorbance with Hidex microplate reader.

In propidium iodide assay, necrotic cell death was measured using propidium iodide (PI, Sigma-Aldrich) and digitonin (Calbiochem, Darmstadt, Germany). PI penetrates damaged cells and binds to DNA, which produces measurable fluorescence. After 24 h exposure, 50 µM PI was added to the wells and fluorescence was measured after 20 min incubation at room temperature in the dark. After that, 160 µM digitonin was added to the cells to damage all the cells, and the fluorescence was measured again after 20 min incubation at room temperature on an orbital shaker. Cell viability was expressed using the following formula: $viability\;\left(\%\right)=100-\frac{F-{blank}_{1}}{F_{max}-{blank}_{2}}*100\%$, where F is fluorescence, blank_1_ is the blank after PI addition, F_max_ is maximum fluorescence after digitonin addition, and blank_2_ is the blank after digitonin addition. Maximum fluorescence represents the total number of cells in each well.

### 2.4. Oxidative Stress Responses

Fluorescent probes dihydroxy ethidium (DHE, Fluka Biochemica, Buchs, Switzerland) and MitoSOX^TM^ Red (Molecular Probes, Invitrogen, Paisley, UK) were used to measure cytoplasmic ROS and mitochondrial superoxide production, respectively, after 24 h dye exposure [7].

DHE is a probe that reacts with intracellular ROS and generates fluorescent oxidation products that can be measured. Exposure medium was removed from the cells and replaced with 10 µM DHE in Hank’s Balanced Salt Solution (HBSS), and the plate was incubated for 30 min at 37 °C, after which the fluorescence (excitation 490 nm/emission 590 nm) was measured with Hidex.

MitoSOX™ is a DHE-derivative that enters mitochondria and reacts with mitochondrial superoxide, forming a fluorescent compound. Similar to the DHE assay, exposure medium was removed from the wells and replaced with 1 µM MitoSOX in HBSS and incubated for 30 min at 37 °C, after which the fluorescence was measured with Hidex.

### 2.5. Genotoxicity

The single-cell gel electrophoresis assay, also known as the comet assay, was used to measure DNA damage after exposure to aglycone extract in HepG2 cells. In the assay, cell samples are mixed with agarose and spread onto a glass microscope slide. Further, the slides were exposed to lysis buffer, thus leaving only nuclei on the slides. The damaged DNA or broken DNA fragments migrate away from the nucleus during electrophoresis and form a tail, resulting in a comet-resembling image. The shape, size, and fragment content of the tail reflect the extent of DNA damage. In this study, DNA unwinding and electrophoresis were carried out under alkaline conditions (pH > 13), which enabled the comet assay to detect DNA double-strand and single-strand breaks. Single-strand breaks are associated with incomplete excision repair sites, DNA-DNA/DNA-protein cross-links and alkali labile sites [23].

The DNA damage was analysed immediately after the exposure, and for the analysis, samples were placed on ice, and the cells were detached from 12-well plates with 1 mL of 0.25% Trypsin in 0.02% EDTA in DPBS. After detaching the cells, samples were suspended in DPBS, and 15 µL (approximately 1.5 × 10^4^ cells) of the cell suspension was mixed in 75 µL of 0.5% low-melting-point agarose. After quick mixing, 80 µL of the suspension was layered onto a microscope slide (pre-coated with a thin layer of 1% normal-melting-point agarose), immediately covered with a coverslip, and kept on ice for 5 min to solidify the agarose. The coverslips were carefully removed, and the slides were immersed in a lysis solution (2.5 M NaCl, 100 mM Na_2_EDTA, 10 mM Tris, 1% Triton X-100, pH 10) and incubated for 1 h at 4 °C in the dark. After incubation, slides were placed in a horizontal electrophoresis unit (Gel Electrophoresis Apparatus, GNA-200, Pharmacia AB, Stockholm, Sweden) for 20 min, allowing the DNA to unwind in the electrophoresis buffer (1 mM EDTA and 300 mM NaOH, pH 13, 4 °C). The electrophoresis was run for 20 min at 24 V. After electrophoresis, the slides were neutralised (3 × 5 min) with Tris buffer (0.4 M, pH 7.5) and fixed in 96% ethanol for 1 min.

For the assay, slides were coded and stained with 20 µg/mL ethidium bromide. The analysis of 100 nuclei per slide was performed with a fluorescence microscope (Axio Imager.A1, Carl Zeiss, Göttingen, Germany) using the comet assay IV version 4.2 (Perceptive Instruments, Haverhill, UK) image analysis software. Olive tail moment (OTM; a measure of tail moment length × a measure of DNA in the tail) was used as the parameter of DNA damage.

### 2.6. Skin Sensitisation

Skin sensitisation potential was measured with the commercial instaCELL^TM^ KeratinoSens^TM^ assay kit (acCELLerate GmbH, Hamburg, Germany). The assay is based on luciferase Nrf2 gene activation in skin keratinocytes, which is considered the second key event in the skin sensitisation Adverse Outcome Pathway (AOP, OECD, 2015).

Briefly, assay-ready keratinocytes were thawed and seeded onto 96-well plates with 10,000 cells per well and pre-incubated for 24 h in a humidified incubator (5% CO_2_ and 37 °C), after which the cells were exposed for 48 h. Then, cell viability was defined by adding 20 µL of resazurin to each well and incubating at 37 °C for 4 h, after which fluorescence was measured with Hidex. Immediately after the measurement, the exposure medium was removed, and the cells were washed once with 50 µL DPBS, after which 50 µL of DPBS and 50 µL of One-Glo^TM^ reagent were added per well. After 20 min incubation at room temperature in the dark, luminescence was measured with an integration time of 1 s/well. Induction result is considered positive if gene activation is increased by 50%.

## 3. Statistical Analysis

Data from in vitro toxicity experiments were analysed using one-way ANOVA followed by Dunn’s post hoc test. GraphPad Prism version 10.0.3 for Windows (GraphPad Prism Inc., San Diego, CA, USA) and the SPSS software version 29.0.0 for Windows (SPSS Inc., Chicago, IL, USA) were used for statistical analyses. Results are expressed as mean ± standard deviation (SD). Differences where *p* < 0.05 were considered statistically significant.

## 4. Results

### 4.1. Dye Isolation Results

**Figure 1:**
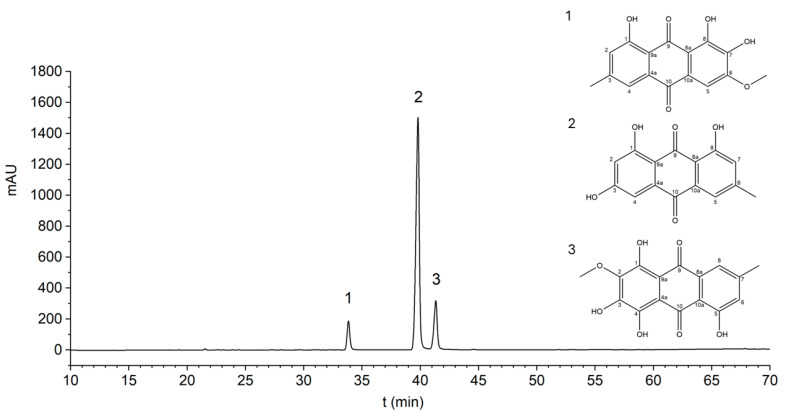
The HPLC chromatogram (detected at 285 nm) of *Cortinarius sanguineus* fraction 1. The numbered peaks correspond to compounds in Table 1. Peak 1 corresponds to dermoglaucin, peak 2 to emodin, and peak 3 to dermocybin. The peak areas do not correspond directly to the abundance of the compound in the extract. The chemical structures of the referred anthraquinones are also presented in the figure.

The result of the chemical analysis of the anthraquinone aglycone extract from C. sanguineus fungus is presented in Table 1, in which the numbers refer to the peaks presented in Figure 1. The fraction of aglycone extract contained emodin and dermocybin as the major compounds and dermoglaucin to a lesser extent. HPLC chromatogram in Figure 1 is detected at 285 nm, and the calculated peak areas for compounds **1**–**3** do not correspond directly to abundance of the compound in the extract. The identification of the compounds was based on their UV-vis spectra (Supplementary Figure S1) and MS-MS data. These data were compared to the scientific literature and previously confirmed studies by Steglich et al. [24], as well as Hynninen, Räisänen, and coworkers’ results [3,4,5,15]. The compound of peak 1 was identified in this study as dermoglaucin because Steglich et al. [24] reported this to be found in *C. sanguineus* but not erythroglaucin, which has the same molecular weight. Further, when examining the shikimate and malonate pathways of anthraquinones biosynthesis, it is more likely that the compound is dermoglaucin, which can be produced by hydrolyzation of C-7 in physcion, a compound also found in *C. sanguineus* [4,5,15,24].

### 4.2. Emodin—Cell Viability and Cytotoxicity

**Figure 2:**
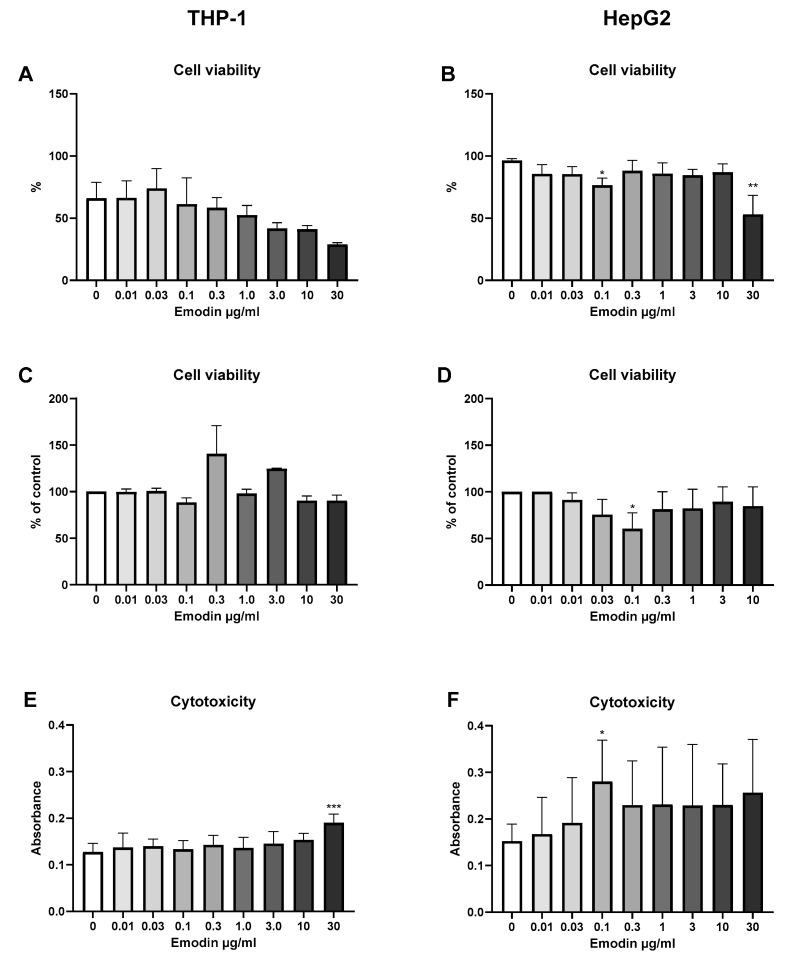
The effect of 24 h emodin exposure on cell viability and cytotoxicity in THP-1 immune cells and HepG2 liver cells. (**A**,**B**) Propidium iodide, (**C**,**D**) MTT, and (**E**,**F**) lactate dehydrogenase (LDH) assays were used to study different types of cytotoxicity. *n* = 3–4; data are shown as mean ± SD. * *p* < 0.05; ** *p* < 0.01; *** *p* < 0.001.

The effect of emodin on cell viability and cytotoxicity was studied with three different viability tests in two cell lines using seven concentrations. As seen in Figure 2B, emodin reduced HepG2 cell viability in high concentrations in the propidium iodide test (*p* < 0.01), and a decreasing trend was also visible in THP-1 cells (Figure 2A). LDH release increased in THP-1 cells at the highest emodin concentration (Figure 2E, *p* < 0.001), indicating cellular membrane damage. No clear dose dependency was observed in other tests in either cell line.

### 4.3. Emodin—Oxidative Stress Responses

**Figure 3:**
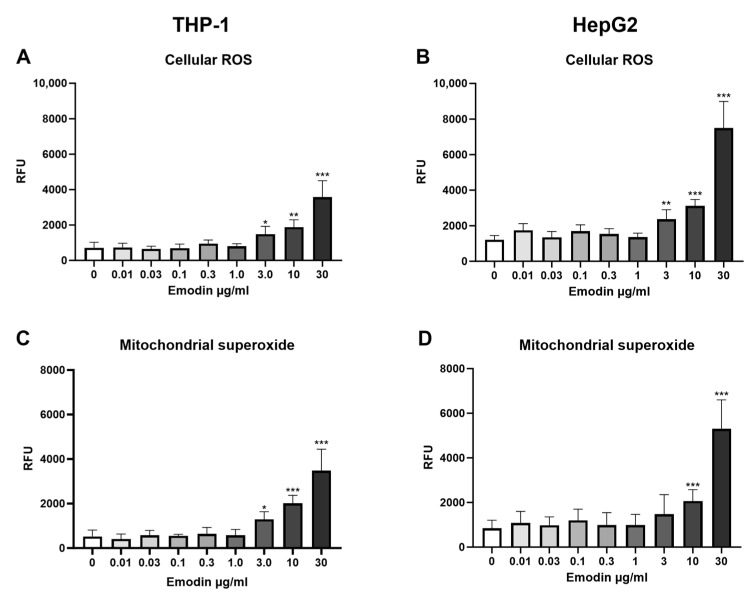
The effect of 24 h emodin exposure on the oxidative stress responses in THP-1 and HepG2 cell lines. (**A**,**B**) intracellular reactive oxygen species (ROS) production, (**C**,**D**) mitochondrial superoxide production. *n* = 3; data are shown as mean ± SD. * *p* < 0.05; ** *p* < 0.01; *** *p* < 0.001. RFU: relative fluorescence unit.

Emodin increased oxidative stress in both cell lines (Figure 3). Intracellular ROS was studied using the DHE assay, and the MitoSOX assay was used to measure mitochondrial superoxide production. In THP-1 cells, 30 µg/mL emodin generated a 5-fold increase in intracellular ROS compared to control (*p* < 0.001, Figure 3A) and a 6.7-fold increase in the mitochondrial superoxide anion production (*p* < 0.001, Figure 3C). Similar changes were observed in HepG2 cells, where intracellular ROS increased 6.2-fold compared to control (*p* < 0.001, Figure 3B), and mitochondrial superoxide production increased by 6.3-fold (*p* < 0.001, Figure 3D).

### 4.4. C. sanguineus Aglycone Extract—Cell Viability and Cytotoxicity

**Figure 4:**
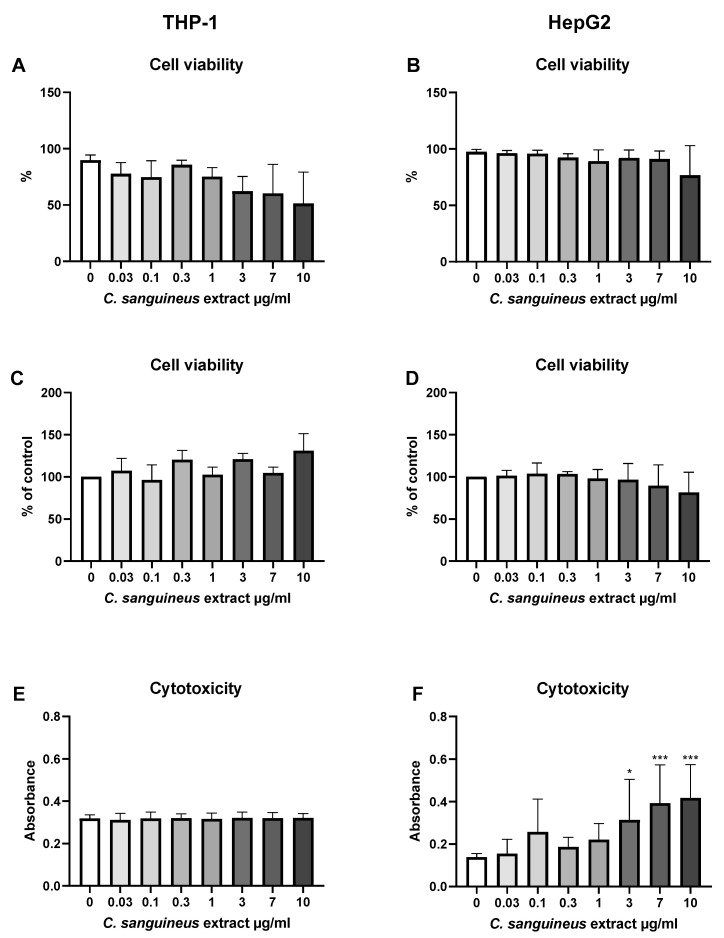
The effect of 24 h *C. sanguineus* aglycone extract exposure on the cell viability and cytotoxicity in THP-1 immune cells and HepG2 liver cells using (**A**,**B**) propidium iodide, (**C**,**D**) MTT, and (**E**,**F**) lactate dehydrogenase (LDH) assay. *n* = 3–4; data are shown as mean ± SD. * *p* < 0.05; *** *p* < 0.001.

Cell viability after *C. sanguineus* aglycone extract exposure was studied with the same viability tests as emodin (Figure 4). The extract affected cell viability only in the LDH test conducted with HepG2 cells, where cell death started to occur at dye concentrations 3 µg/mL (*p* < 0.05), 7 µg/mL (*p* < 0.001), and 10 µg/mL (*p* < 0.001) (Figure 4F).

### 4.5. C. sanguineus Aglycone Extract—Oxidative Stress Responses

**Figure 5:**
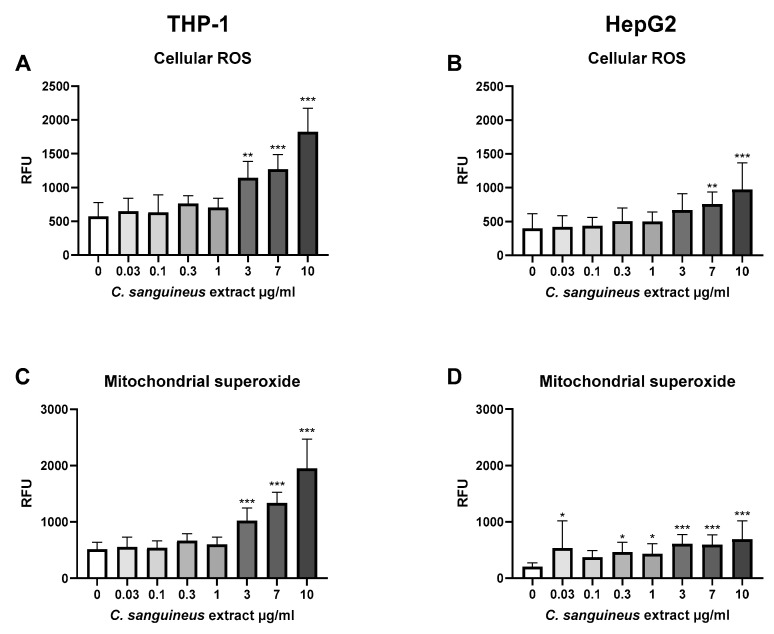
The effect of 24 h *C. sanguineus* aglycone extract exposure on (**A**,**B**) cellular ROS and (**C**,**D**) mitochondrial superoxide in THP-1 immune cell line and HepG2 liver cells. *n* = 3; data are shown as mean ± SD. * *p* < 0.05; ** *p* < 0.01; *** *p* < 0.001. RFU: relative fluorescence unit.

*C. sanguineus* aglycone extract caused a dose-dependent increase in the production of both mitochondrial superoxide and intracellular ROS in both cell lines (Figure 5). In THP-1 cells, intracellular ROS increased 3.2-fold compared to control (*p* < 0.001, Figure 5A), and the effect was seen already at 3 µg/mL (*p* < 0.001), whereas mitochondrial superoxide production increased 3-fold at 10 µg/mL extract exposure compared to control (*p* < 0.001, Figure 5C).

In HepG2 cells, the dose–response relationship was not as clear as in THP-1 cells, but the highest 10 µg/mL concentration showed a 3.4-fold increase in mitochondrial superoxide generation (*p* < 0.001, Figure 5D) and a 2.4-fold increase in the intracellular ROS generation (*p* < 0.001, Figure 5B) compared to control.

### 4.6. C. sanguineus—Genotoxicity Results

**Figure 6:**
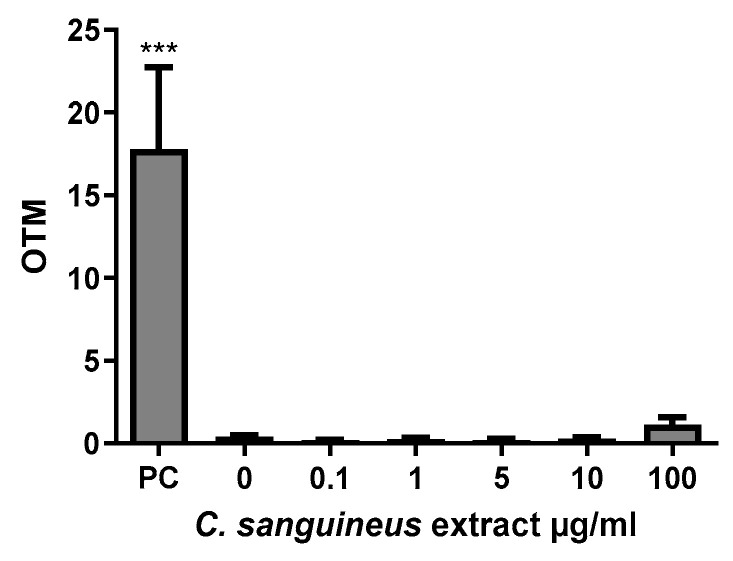
The effect of 24 h *C. sanguineus* aglycone extract exposure on the DNA damage of HepG2 liver cells in the comet assay. OTM: Olive Tail Moment; PC: positive control; methyl methanesulfonate: 40 µg/mL. *n* = 3; data are shown as mean ± SD. *** *p* < 0.001.

No signs of DNA damage were detected in the comet assay after exposure to 0.1–100 µg/mL aglycone extract in HepG2 cells, as presented in Figure 6.

### 4.7. Skin Sensitisation Potential

**Figure 7:**
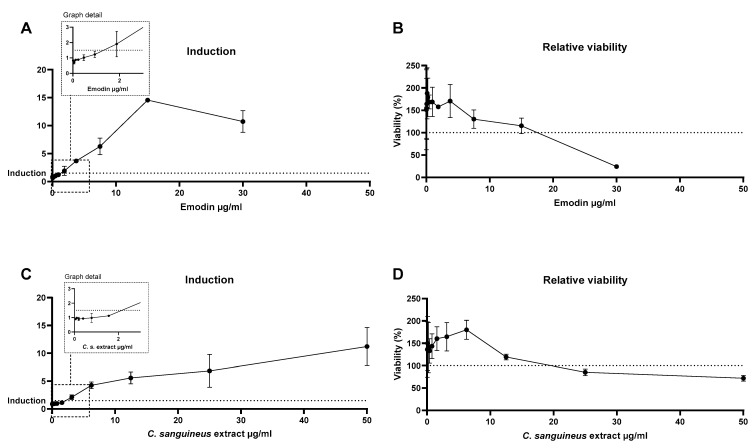
The skin sensitisation assay results depicting the Nrf2 gene induction levels and relative keratinocyte viability after 48 h exposure to emodin or *C. sanguineus* aglycone extract. (**A**) shows the result of emodin induction and its effect on viability (**B**). Panel (**C**) shows the induction after the extract exposure and (**D**) cell viability after exposure to the extract. The extract precipitated at 100 µg/mL, causing total cell death and the result was omitted from the figure (**D**). The induction points for emodin and the extract are highlighted in the graph details. Data are shown as mean ± SD; *n* = 2.

Both *C. sanguineus* aglycone extract and emodin exhibited skin sensitisation potential in the KeratinoSens assay (Figure 7A,C). According to the assay criteria, induction occurs when a 1.5-fold gene induction limit is exceeded. This occurred for emodin at 2.0 µg/mL concentration and for the extract at 2.4 µg/mL, as shown in the graph details of Figure 7A,C. In the resazurin assay, it was seen that both exposures decreased cellular viability (Figure 7B,D). In the cells exposed to emodin, the cell viability decreased by 76% at the highest concentration used (30 µg/mL, Figure 7B). *C. sanguineus* aglycone extract also decreased the viability by approximately 30% compared to control at doses 25 and 50 µg/mL. At 100 µg/mL dose, the extract precipitated, causing a total cell death. That result has been omitted from Figure 7.

## 5. Discussion

*Cortinarius sanguineus* fungus contains fifteen distinct anthraquinones, and from those, many have the potential to be used as biobased dyes in various applications, such as textiles and biodegradable food packaging [3,7,25]. In this study, we examined, for the first time, the toxicity and skin sensitisation potential of the *C. sanguineus* aglycone extract, which contains the most abundant anthraquinones of *C. sanguineus* fungus, emodin and dermocybin. In addition, its single dye component emodin was assessed in the same experimental setting, similar to previously studied dermocybin [7]. Finally, the toxicity of individual dyes of *C. sanguineus* was compared with the data of the anthraquinone aglycone extract.

*C. sanguineus* aglycone extract caused cytotoxicity in HepG2 liver cells after exposure to the highest 3–10 µg/mL concentrations, indicated by dose-dependent LDH release. In the resazurin assay, 25 and 50 µg/mL concentrations also slightly decreased cell viability, but no effects were detected with lower doses. On the other hand, no effects were seen in the MTT and propidium iodide assays in HepG2 cells, and the viability of THP-1 cells was not affected. It seems that the aglycone extract is cytotoxic only in high doses and that the mechanism of cytotoxicity is necrotic.

Moreover, emodin exposure increased LDH release in THP-1 cells, but only at the highest 30 µg/mL dose, similar to the resazurin assay. In addition, 30 µg/mL emodin decreased cell viability in the propidium iodide test in HepG2 cells and in THP-1 cells but without statistical significance. Furthermore, no effects were observed in the MTT assay after 24 h exposure to emodin in either cell line. However, other studies have reported decreased viability in MTT assay after emodin exposure: 50–100 µM (14–27 µg/mL) emodin decreased HepG2 viability down to 40% in a 48 h study [26] and Qu et al. [27] observed a dose-dependent decrease in human T-cell viability after 24–72 h exposure, and the decrease was already visible after 24 h. Further, Brkanac and colleagues exposed human peripheral blood lymphocytes (HPBL) to emodin, which decreased their viability at 150 µg/mL and higher concentrations when stained with ethidium bromide [28]. Our results are in line with other studies on emodin, as we saw that emodin exhibited necrotic cytotoxicity at high concentrations in some assays, but the results were variable depending on the study setting, as seen in previous studies.

In the present study, we observed dose-dependent effects in the cytosolic ROS and mitochondrial superoxide production after both emodin and extract exposure, but only a minor decrease in cell viability. Brkanac and colleagues [28] achieved a similar result with emodin, where it induced cytosolic ROS production after 25 µg/mL concentration, but viability was affected only after 150 µg/mL exposure.

When comparing the present results with our previous study on dermocybin, which is the second most common compound in *C. sanguineus* after emodin, the results are interesting. In our earlier study, similarly to the aglycone extract, dermocybin increased LDH release in HepG2 cells but not in THP-1 cells. Additionally, 7 µg/mL dermocybin exposure reduced the HepG2 cell viability by 23% in the MTT viability assay [7]. Dermocybin also increased mitochondrial superoxide production, but no effects were detected in cytosolic ROS levels [7]. However, both the aglycone extract and emodin increased cytosolic ROS levels, which could indicate that emodin mediates these effects in the extract, as this effect was not seen in dermocybin.

We did not observe DNA damage in the comet assay in HepG2 liver cells exposed to *C. sanguineus* aglycone extract. Similarly, Brkanac et al. [28] used emodin-containing tree bark extract to expose human peripheral blood cells and observed DNA damage at 500–1500 µg/mL levels, but emodin alone caused it at 200 µg/mL and higher concentrations. The effect did not change when the S9 mix was included. Additionally, Müller et al. [29] have tested 55 µM emodin in murine cells without genotoxic effects. However, increased DNA damage has been observed in comet assay after 20 µg/mL emodin exposure [30]. Dermoglaucin, a minor component of the aglycone extract, showed some potential for mutagenicity in the presence of S9 in a study by Brown and Dietrich [31], but no other studies have been conducted, to our knowledge.

We were the first to show that skin sensitisation potential occurred both after emodin and *C. sanguineus* aglycone extract exposure. The induction concentrations of the exposures were similar, circa 2–2.5 µg/mL. This is noteworthy since emodin is widely used, and it has been previously considered an anti-allergenic substance, among other beneficial health effects [32,33]. Dermocybin was not a skin sensitiser according to our study [7]. Therefore, it is likely that emodin causes the induction also in the extract. Nevertheless, as this test studies only one molecular event, the OECD recommends its confirmation with another sensitisation assay [34]. As textiles are likely the most common targets for dyeing, the individuals applying the dyes and wearing textiles dyed with the whole *C. sanguineus* extract should be aware of the risk of its allergenicity.

Since the small-scale dyers and crafters are potentially exposed to traditionally used *C. sanguineus* dye extract, as such, we studied the mixture of the most abundant anthraquinones of *C. sanguineus*. In the future, it would be intriguing to determine the toxicity of the dye extract of another common *Cortinarius* fungus used in dyeing, *C. semisanguineus*. According to Steglich et al. [24], it contains considerably less emodin than *C. sanguineus*. This would make it a more promising candidate for use as a dye from a safety point of view. However, even though the raw extract of *C. sanguineus* contains dermoglaucin as a minor component, it is the third largest component in the aglycone extract, and consequently, it is a clear limitation of the study that we did not evaluate the toxicity of dermoglaucin.

Altogether, this research contributes to the growing interest in using natural colourants instead of synthetic dyes. However, when exploring natural colourants as alternatives to synthetic dyes, it is important to assess their potential safety hazards to decrease the likelihood of adverse effects on humans or the environment.

## 6. Conclusions

We are the first to study the toxicity of *C. sanguineus* aglycone extract, which contains emodin and dermocybin as its two main components, and to show that the extract, as well as emodin, has skin sensitisation potential. In addition, we studied the toxicity of emodin, and together with our previously published results on dermocybin, we evaluated the in vitro toxicity of the *C. sanguineus* extract. Our results show that *C. sanguineus* aglycone extract caused harmful effects in human cells. It is possible that many of these effects are mediated by its major component, emodin. This research points out that natural dyes are not necessarily safe, and the community using natural dyes should be informed so that they can protect themselves. *C. semisanguineus*, another fungus that contains anthraquinone dyes, which contains considerably less emodin, would be an interesting comparison for toxicological studies. In addition, the toxicity of dermoglaucin warrants future studies.

## Figures and Tables

**Table 1:** Anthraquinone aglycones in *Cortinarius sanguineus* extract fraction 1. Sample’s concentration at 1 mg/mL in MeOH, MS *m*/*z* 50–750. sh = shoulder. The numbering of the peaks refers to the HPLC chromatogram shown in Figure 1. The calculated peak areas for compounds **1**–**3** do not correspond directly to abundance of the compound in the extract. References: [15,24].

No.	Rt (min)	*λ*_max_ (nm)	*A*_430_/*A*_254_	*A*_430_/*A*_285_	MS(−) *m/z* (%)	MS/MS(−) *m/z* (%)	MS(+) *m/z* (%)	Molecular Formula (Mass)	Proposed Compound	Peak Area % *
**1**	33.8	224, 284, 432	0.81	0.41	621 (65) [2M − 2H + Na]^−^, 299 (100) [M − H]^−^	299: 284 (100) [M − H − CH_3_]^−^	339 (11) [M + K]^+^, 323 (33) [M + Na]^+^, 302 (100) [M + 2H]^+^	C_16_H_12_O_6_ (300.26)	Dermoglaucin	>5
						621: 299 (100) [M − H]^−^, 284 (41) [M − H − CH_3_]^−^, 256 (13) [M − H − CH_3_ − CO]^−^				
**2**	39.6	228, 254, 268, 288, 440	0.61	0.54	269 (100) [M − H]^−^	269: 225 (100) [M − H − CO_2_]^−^	271 (100) [M + H]^+^	C_15_H_10_O_5_ (270.24)	Emodin	>10
**3**	41.2	226, 263, 276sh, 306sh, 462sh, 485, 514sh	0.35	0.46	653 (18) [2M − 2H + Na]^−^, 315 (100) [M − H]^−^	315: 300 (100) [M − H − CH_3_]^−^	355 (13) [M + K]^+^, 339 (27) [M + Na]^+^, 318 (100) [M + 2H]^+^	C_16_H_12_O_7_ (316.26)	Dermocybin	>10
						653: 315 (100) [M − H]^−^, 300 (33) [M − H − CH_3_]^−^, 272 (6) [M − H − CH_3_ − CO]^−^				

* Calculated from the 285 nm chromatogram.

## Data Availability

The raw data supporting the conclusions of this article will be made available by the authors on request.

## Supplementary Materials

The following supporting information can be downloaded at: https://www.mdpi.com/article/10.3390/jof10060369/s1, Figure S1: The UV-vis spectra of (A) dermocybin, (B) dermoglaucin, and (C) emodin.
