# Supplementary material for: In Vitro Toxicity Assessment of Cortinarius sanguineus Anthraquinone Aglycone Extract
Source: J Fungi (Basel). 2024 May 21;10(6):369. doi: 10.3390/jof10060369 (PMC11204901; doi:10.3390/jof10060369)
Supplement: Supplementary file 1 [file jof-10-00369-s001.zip › jof-2978453-supplementary.pdf]

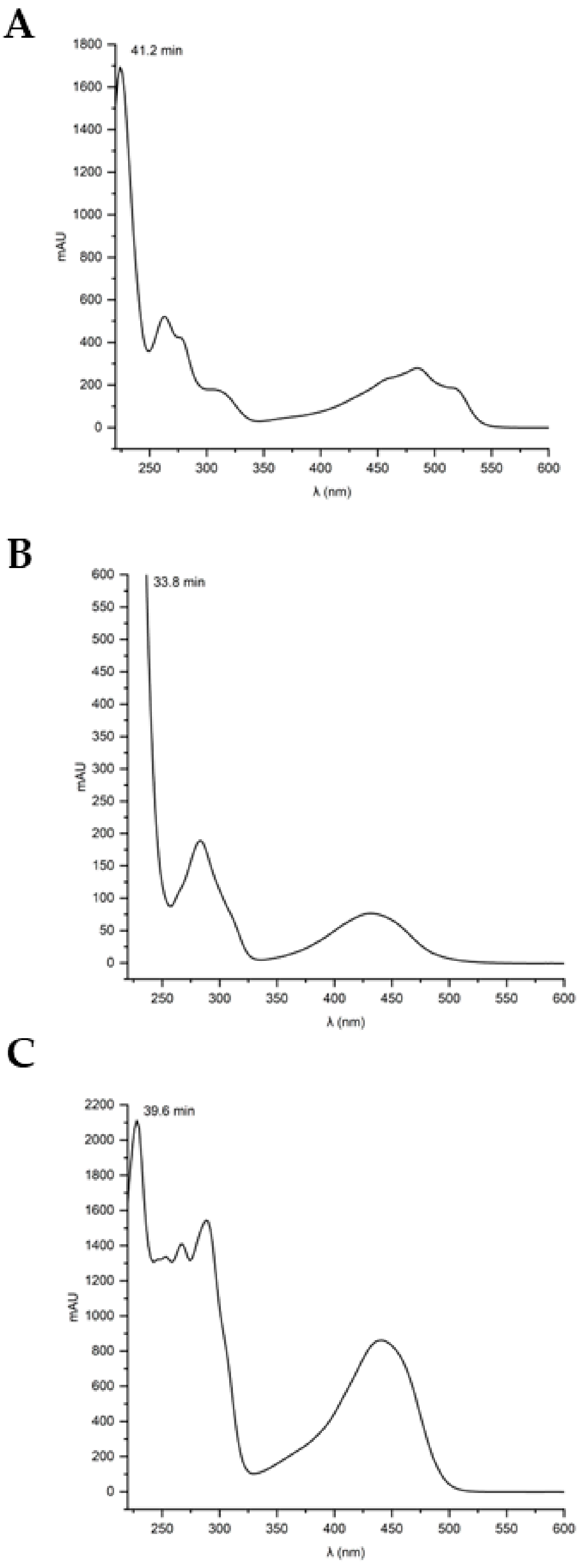

Supplementary Figure S1. *The UV-vis spectra of A) dermocybin, B) dermoglaucin, and C) emodin. The minutes in the figures represent the retention time. mAU = milli-absorbance unit.*
